# Possibilities of K-Value Determination for Active Admixtures with Respect to Durability

**DOI:** 10.3390/ma18102227

**Published:** 2025-05-12

**Authors:** Petr Šperling, Rudolf Hela, Adam Hubáček, Tereza Stará, Richard Dvořák

**Affiliations:** 1 Faculty of Civil Engineering, Institute of Technology of Building Materials and Components, Brno University of Technology, Veveří 331/95, 602 00 Brno, Czech Republicadam.hubacek@vut.cz (A.H.); tereza.stara@vut.cz (T.S.); 2 Faculty of Civil Engineering, Institute of Construction Testing, Brno University of Technology, Veveří 331/95, 602 00 Brno, Czech Republic; richard.dvorak@vut.cz

**Keywords:** k-value, activity index, supplementary cementitious materials, fly ash, durability

## Abstract

This paper discusses the possibility of determining k-values for active admixtures concerning durability factors such as the depth of penetration of water under pressure and the depth of carbonation of cement mortars with fly ash. The k-value considers the use of active admixtures in concrete when calculating the water/cement ratio and the equivalent amount of binder. Currently, only the effect of the active admixture on the compressive strength of concrete and cement mortars is considered when determining the k-value, but not the effect of the active admixture on durability. To account for the influence of durability factors on the determination of the k-value, the mathematical functions of the property, dependent on the water/cement ratio and the age of the cement mortar, were constructed using regression analysis. From the determined functions, it was then possible to use an optimisation problem to determine the k-value so the difference between the actual measurement and calculated depth of pressure water seepage or carbonation was as small as possible. A high coefficient of determination of 0.9855 was achieved for the pressure water seepage depth function, but the coefficient of determination for the carbonation depth was lower.

## 1. Introduction

Concrete is the most commonly used building material and has a significant impact not only on the overall cost of construction but also on its environmental impact. In the production of concrete, cement is the basic raw material. It is formed by firing limestone and other rocks. This firing produces a high amount of CO_2_ (approximately 0.7 t per tonne of Portland cement) [1,2]. For this reason, there is now an increasing pressure to reduce the amount of CO_2_ emitted into the atmosphere. At the same time, there is a significant increase in the price of emission allowances, which increases the cost of concrete and, consequently, of buildings. The price of one tonne of CO_2_ emitted into the atmosphere was only EUR 6 in 2016, and in 2025, it will already be about EUR 80 per one tonne of CO_2_ emitted. In particular, the price of cement and its environmental impact are the main reasons for using alternative binders or active admixtures from secondary raw materials to partially replace cement for concrete production [3].

Pozzolans, such as the natural kind, or energy by-products, such as power plant fly ash, can be used as partial cement substitutes. Another option comprises latent hydraulic substances such as finely ground granulated blast furnace slag. An interesting material is micro-ground limestone, which, although not pozzolanic or latent-hydraulic in nature, is partly active but mainly acts as a passive, fine-grained admixture. In general, admixtures affect the properties of concrete in both the fresh and hardened state. In the fresh state, they primarily affect the consistency; in the hardened state, they affect the resulting mechanical properties, volume changes, and concrete durability [4,5,6].

The principles of using active admixtures in concrete as partial replacements for cement are captured by the concept of the k-value. This concept is a prescribed method for assessing the quality and dosage limits of active admixtures for inclusion in calculating the water/cement ratio. The concept is based on comparing the compressive strength of concrete without an active admixture to that of concrete in which part of the cement has been replaced by an active admixture. The k-value concept allows the use of active admixtures if [7,8] the following conditions are met:

The water/cement ratio is replaced by the equivalent water/cement ratio water/(cement + k × admixture).

The weight of the cement + k × admixture is greater than the minimum amount of cement required for the exposure class according to EN 206+A2 [8].

The effects of active admixtures on the properties of concrete depend on the nature of the individual materials, the age of the concrete, the external conditions, etc. In order to consider these aspects when designing the concrete composition, the k-value concept uses the relationship between the water/cement ratio and the compressive strength according to the Abrams law [9,10].

In the European standard EN 206+A2, the concept of the k-value is described in very general terms, and the possibility of its precise determination is not mentioned. For example, EN 206+A2 specifies a k-value of 0.4 for fly ash for use up to a fly ash/cement ratio of 0.33 with CEM I cement and up to a ratio of 0.25 with CEM II cement, regardless of the age of the concrete or the amount of active admixture used. The results obtained in [11] and [12] have demonstrated that the k-values determined from the relationship between the water/cement ratio and compressive strength vary for different cement replacements of active admixture and concrete ages. To determine the k-value, reference is made to CEN/TR 16639, which provides a procedure for determining the k-value based on compressive strength only and does not consider the effects of active admixtures on the durability of concrete [8,9]. The literature mentions the possibility of determining the k-value for the rate of concrete carbonation and the resistance to chloride penetration [13,14]. In the absence of a general description of the relationship between the water/cement ratio and carbonation depth, an inverse approach was used for this dependence in [14]. Instead of directly determining the k-value, the k-values determined from the compressive strength were used for the carbonation resistance, the suitability of which was assessed based on the relationship between the carbonation coefficient and the equivalent water/cement ratio. This procedure was also used for chloride resistance but proved unsuitable. Therefore, in this case, the author established a relationship between the water/cement ratio and diffusion coefficients based on the results obtained, which were utilised to determine the k-values of the active admixtures used.

## 2. Materials and Methods

In the experimental section of the study, formulations were designed and subsequently utilised to mix cement mortars. The mortars were mixed using Portland cement CEM I 42.5 R from the Heidelberg Mokrá (Mokrá, Czech Republic) cement plant as reference cement mortars. The reference cement mortars were mixed with three different water/cement ratios of 0.43, 0.5, and 0.53. In addition, cement mortars were mixed with 10%, 20%, and 30% cement replacement with Opatovice fly ash. Silica sand with fractions of 0.1–0.6 mm, 0.6–1.2 mm, and 1–4 mm was used as filler. Physical and chemical composition is in Table 1 and Table 2. The aggregate grain size curve was designed to match the grain size curve of standard sand as closely as possible according to EN 196-1 [15]. The cement mortar mix proportions used are shown in Table 3.

The consistency of the cement mortars was determined in the fresh state using a shaking table according to EN 1015-3 [16]. In the hardened state, the compressive strength on a prism of 40 × 40 × 160 mm was tested according to EN 196-1, and the depth of pressure water penetration according to EN 12390-8 [17] and the depth of carbonation according to EN 13295 [18] were tested at the ages of 7, 28, 60, and 90 days on all cement mortar mix proportions.

The depth of penetration of water under pressure was, on cubes with an edge of 150 mm, tested. The samples were exposed to water for 72 h. The water pressure was 0.5 MPa. At ages of 7, 28, 60, and 90 days, the samples tested for depth of carbonation were placed in a chamber with 3% CO_2_, 60% humidity, and 20 °C. Samples were stored for 56 days in the carbonation chamber.

Based on the obtained compressive strength results, an activity index was determined according to EN 450-1 [19]. Furthermore, based on the relationship between the water/cement ratio and the compressive strength, k-values for compressive strength were determined for each age and replacement of cement with fly ash.

Determination of the k-value for fly ash was made based on the relationship between the water/cement ratio and compressive strength using the following formula [11]. Based on Abram’s law, it is possible to calculate compressive strength of concrete. Abram’s law determines the relationship between water/cement ratio and compressive strength, and with some mathematical adjustment it is possible to calculate k-value for active additions [11].

(1) $$k=\frac{w_{a}\cdot \left(\frac{f_{ca,t}}{K}+a_{t}\right)-c}{a_{f}}$$

Here, k is the k-value [-]; w_a_ is the amount of water [kg/m^3^]; f_c_, a, t is the compressive strength [MPa] at age t [days] and the amount of admixture a [%]; at is the coefficient depending on the age of the samples [-]; c is the amount of cement [kg/m^3^]; and a_f_ is the amount of fly ash [kg/m^3^].

To determine the k-value for the durability aspects (in this case, the depth of penetration of water under pressure and the depth of carbonation), age- and water/cement ratio-dependent functions were developed for each property. These functions were created using regression analysis, with the property as the dependent variable and age and water/cement ratios as the independent variables. Predominantly polynomial dependencies (e.g., Equation (2)) were chosen for the preparation of these functions so the resulting dependencies would have a high coefficient of determination (R2) or would describe the course of the dependency as accurately as possible.

(2) $$DA=\beta_{0}+\beta_{1}\cdot t+\beta_{2}\cdot w+\beta_{3}\cdot t^{2}+\beta_{4}\cdot w^{2}+\beta_{5}\cdot w\cdot t$$

Here, DA is the durability aspect; β0, β1, β2, β3, β4, and β5 are empirically determined coefficients [-]; t is the age [days]; and w is the water/cement ratio [-].

After determining the dependencies of the durability aspects, the k-value was determined using an optimisation problem. To calculate the k-value for each durability aspect, the water/cement ratio w was replaced by (3) in the above-mentioned dependence (2).

(3) $$w=\frac{w_{a}}{c+k\cdot a_{f}}$$

Thus, a modified equation was obtained (2):

(4) $$DA=\beta_{0}+\beta_{1}\cdot t+\beta_{2}\cdot \left(\frac{w_{a}}{c+k\cdot a_{f}}\right)+\beta_{3}\cdot t^{2}+\beta_{4}\cdot {\left(\frac{w_{a}}{c+k\cdot a_{f}}\right)}^{2}+\beta_{5}\cdot \left(\frac{w_{a}}{c+k\cdot a_{f}}\right)\cdot t$$

The objective function of the optimisation problem was chosen to be the absolute value of the difference between the actual measured property of the cement mortar and the calculated property from the dependence of the property on the age and water/cement ratio (5). The k-value was chosen as the parameter of this function:

(5) $$f_{\left(k\right)}=\left|{DA}_{C}-{DA}_{R}\right|$$

Here, f(x) is the objective function of the optimisation problem, DAC is the calculated value of the property according to Equation (4), and DAR is the actual measured value of that aspect of durability.

**Figure 1:**
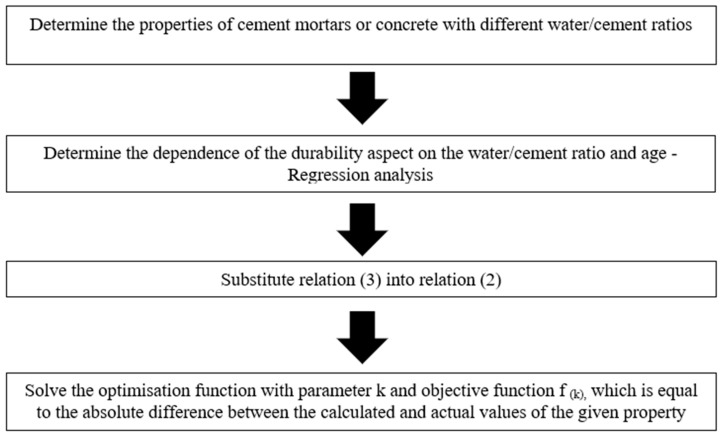
Schematic of determining the k-value using durability.

After selecting the parameters and the objective function, the k-values were determined so that the absolute difference between the calculated value of the given property and the real measured value of this property was as small as possible. This procedure is shown in Figure 1.

## 3. Results

This section presents the results of determining the properties of cement mortars. First, the properties of cement mortars in the fresh state are given, followed by the results of the hardened mortars: the compressive strength, depth of penetration of water under pressure, and carbonation depth. This section concludes with the dependencies of the compressive strength, depth of penetration of water under pressure, and carbonation depth of the different cement mortars. Their coefficients of determination and a detailed description of the determination of the k-value for each property using the procedure described in the previous section are also given.

### 3.1. Consistency of Fresh Cement Mortars

**Figure 2:**
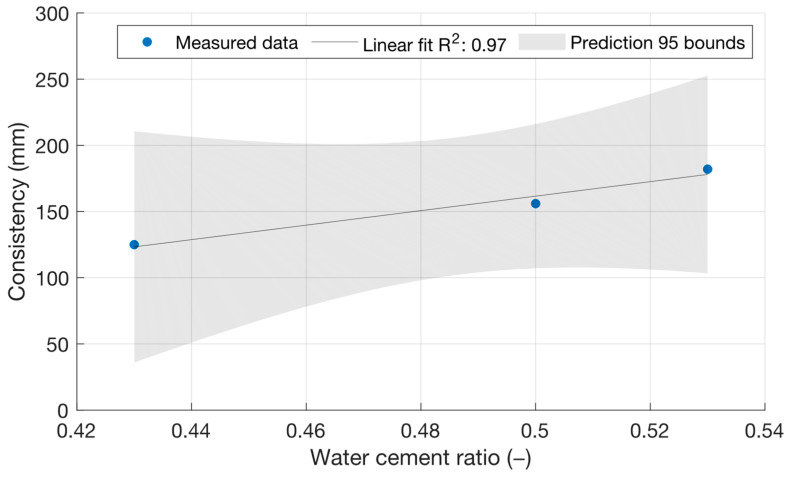
Consistencies of reference fresh mortars with different water/cement ratios.

**Figure 3:**
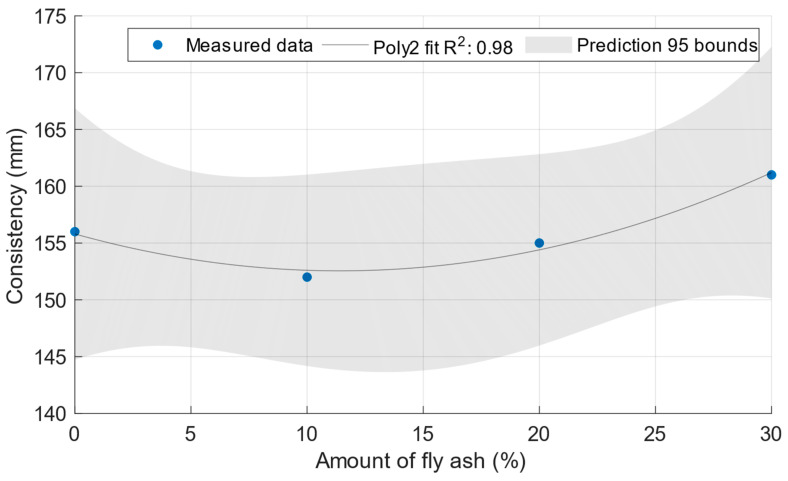
Consistencies of fresh mortars with the fly ash Opatovice.

Figure 2 shows that, as expected, the cement mortar spillage increased with an increasing water/cement ratio up to a value of 182 mm. Figure 3 shows the consistencies of the cement mortars with power plant fly ash with a constant water/cement ratio of 0.5. In this case, there was no significant difference between the consistencies of the cement mortars.

### 3.2. Properties of Hardened Cement Mortars

**Figure 4:**
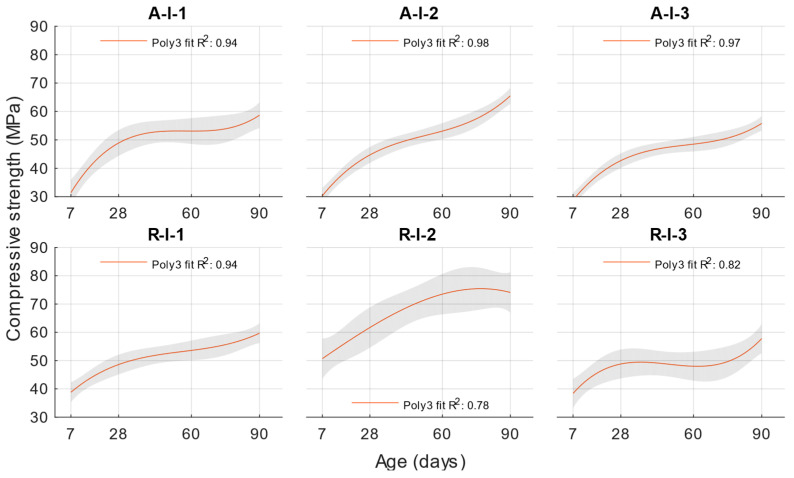
Compressive strength.

In the hardened state, the compressive strengths were first determined. Based on these results, an activity index was then calculated according to EN 450-1 for the individual replacements and the age of the cement mortar with fly ash. The results of the compressive strengths are shown in Figure 4.

For cement mortars with different water/cement ratios, the lowest compressive strengths were obtained with a water/cement ratio of 0.53, and the compressive strengths increased with a decreasing water/cement ratio. At ages of 7 and 28 days, lower compressive strengths were achieved for cement mortars with fly ash than the reference formulation with the same water/cement ratio. However, at ages of 60 and 90 days, the compressive strengths and activity indices increased up to 100%.

For cement mortars with different water/cement ratios, the depth of penetration of water under pressure was lowest for cement mortar with a water/cement ratio of 0.43 and highest for cement mortar with a water/cement ratio of 0.53. For the cement mortar with Opatovice power plant fly ash, the depth of penetration of water under pressure at an age of 7 days was higher than for the reference formulation with the same water/cement ratio. However, the samples with higher cement replacement using fly ash had the worst results. As the age of the specimen increased, the depth of penetration of water under pressure decreased, and at ages of 60 and 90 days, it reached the same or lower values than the cement mortar without fly ash.

The course of the depth of carbonation was the same as the course of the depth of penetration of water under pressure. Again, the depth of carbonation increased with the increased water/cement ratio, as well as with the increased amount of fly ash in the cement mortar.

At the age of 7 days, there was a significant difference in the depth of carbonation from 1.17 mm for the reference cement mortar with a water/cement ratio of 0.5 to 7.42 mm for the cement mortar with 30% power plant fly ash by weight of the original cement. At the age of 28 days, this difference decreased, and there was an increase in the depth of carbonation of 2.08 mm for the A-I-30 recipe. Further, at ages of 60 and 90 days, the total difference in depth of carbonation was less than 1 mm.

### 3.3. Determination of the K-Value of Power Plant Fly Ash

The k-value of power plant fly ash was first determined using Formula (1). To assess whether the k-value could also be determined using regression analysis using the procedure described in Section 2, this determination was also carried out for the compressive strengths of cement mortars with fly ash.

The following function was determined for compressive strength using regression analysis, as shown in Figure 4. The coefficient of determination of this function was 0.99.

(6) $$f_{c}\left(w,t\right)=417.66-1380\cdot w+0.2207\cdot t+1241\cdot w^{2}+0.2805\cdot w\cdot t-0.0012\cdot t^{2}$$

**Figure 5:**
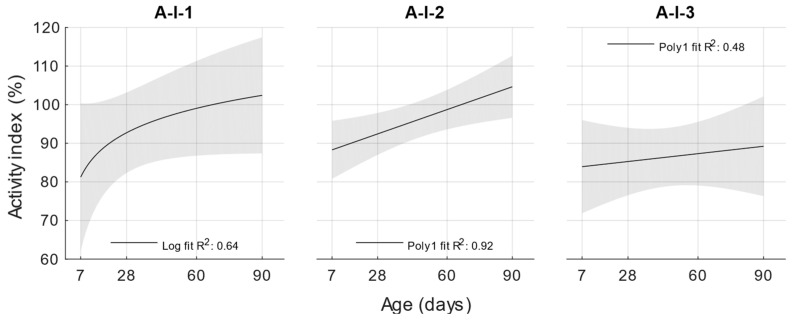
Activity index.

**Figure 6:**
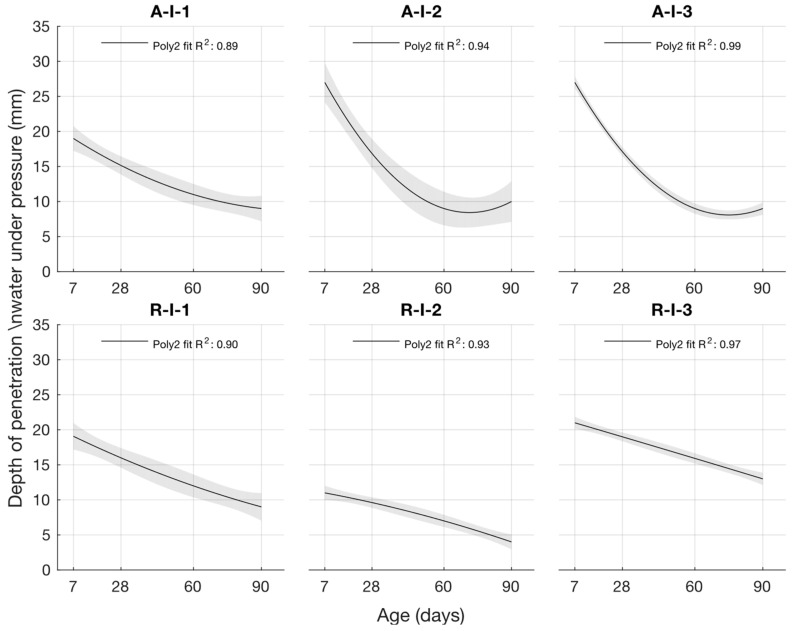
Depth of penetration water under pressure.

**Figure 7:**
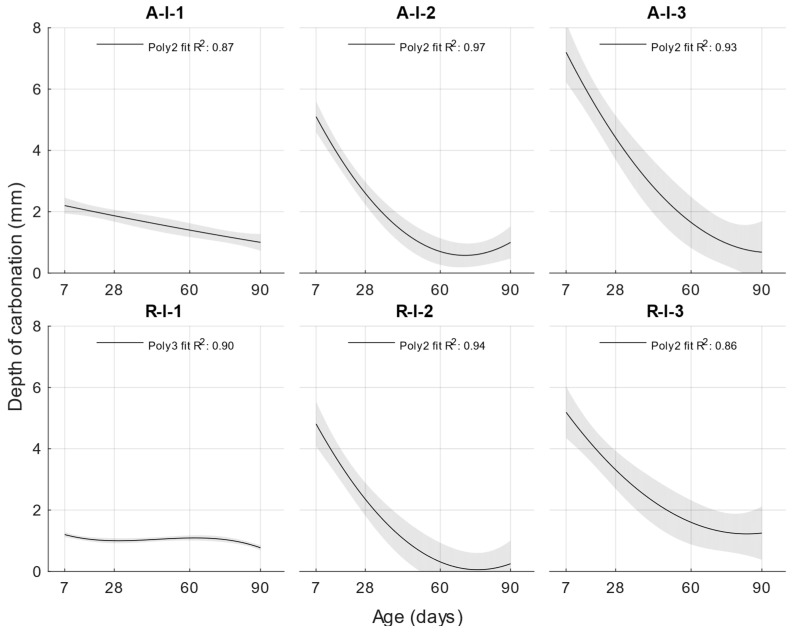
Depth of carbonation.

The coefficient of determination R^2^ = 0.9404 was determined for this function. From this, it can be concluded that the compressive strength function is sufficiently accurate for calculating the compressive strength of cement mortars with water/cement ratios of 0.5, 0.43, and 0.53. To better see the difference between the actual compressive strength values and the calculated values, Figure 5, Figure 6 and Figure 7 show these values for each water/cement ratio, and the numerical difference is provided in Table 4.

Figure 5, Figure 6 and Figure 7 show no significant deviations between the actual and calculated compressive strength values for the water/cement ratios shown. These values are compiled in Table 4, where the differences of the residuals, i.e., the differences between the measured compressive strength and the one calculated utilising the function used, are shown. In particular, the largest deviations occurred for the compressive strength results at ages of 7 and 28 days. It can be concluded that for a given age, the calculated k-values will not completely match those determined using Formula (1).

The same calculation can be performed for the strength of cement mortars with fly ash. Substituting Formula (3) into the compressive strength function Equation (6), the compressive strength of cement mortar with fly ash can be calculated after substituting the values. In this calculation, the k-value was used in Formula (3), which was calculated using the optimisation problem; the procedure is presented in Section 2. The following graphs and Table 5 show the differences between the calculated and measured compressive strength values for the different cement replacements with fly ash.

**Figure 8:**
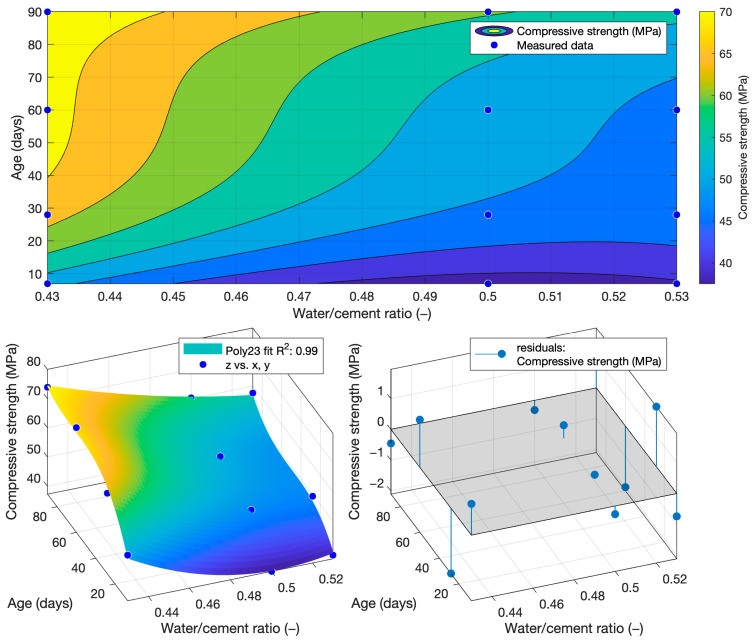
Function of compressive strength.

**Figure 9:**
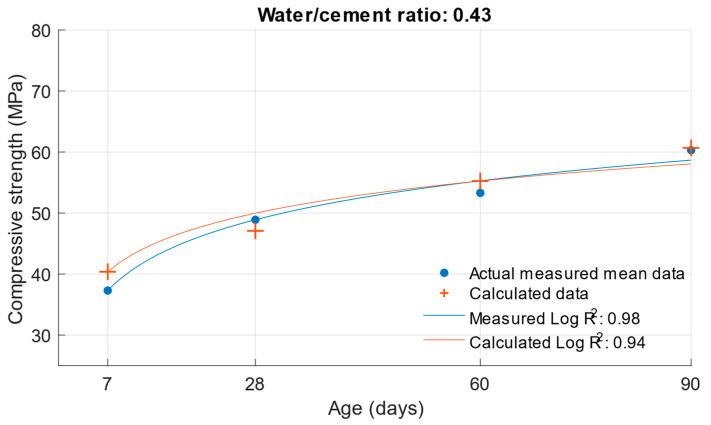
Difference between measured and calculated compressive strength with a water/cement ratio of 0.43.

**Figure 10:**
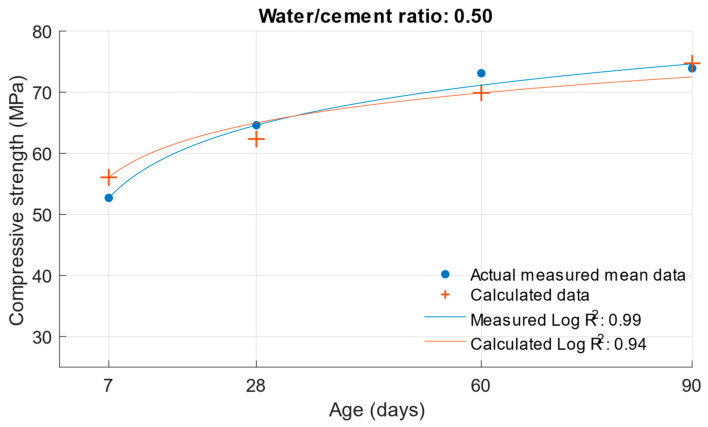
Difference between measured and calculated compressive strength with a water/cement ratio of 0.5.

**Figure 11:**
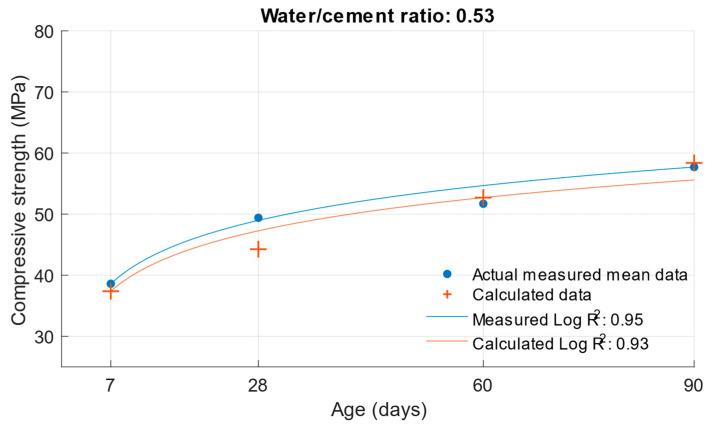
Difference between measured and calculated compressive strength with a water/cement ratio of 0.53.

**Figure 12:**
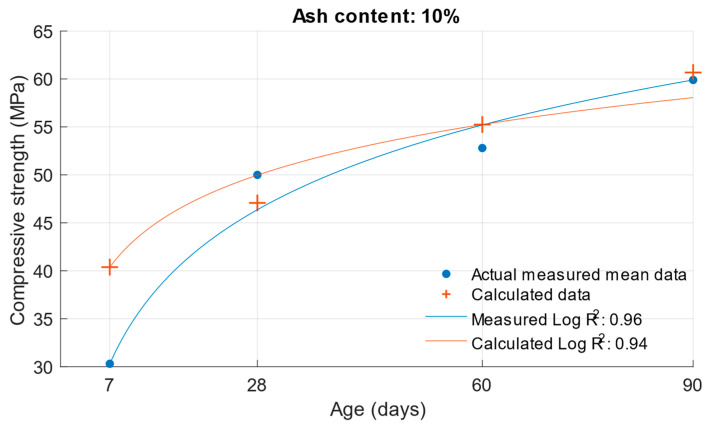
Difference between measured and calculated compressive strength with 10% fly ash.

**Figure 13:**
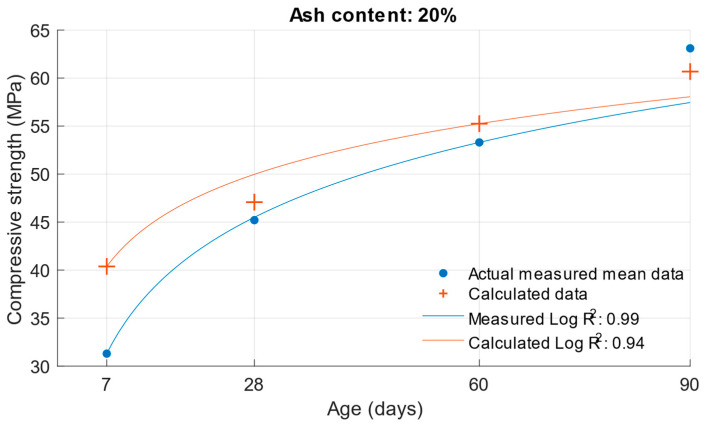
Difference between measured and calculated compressive strength with 20% fly ash.

**Figure 14:**
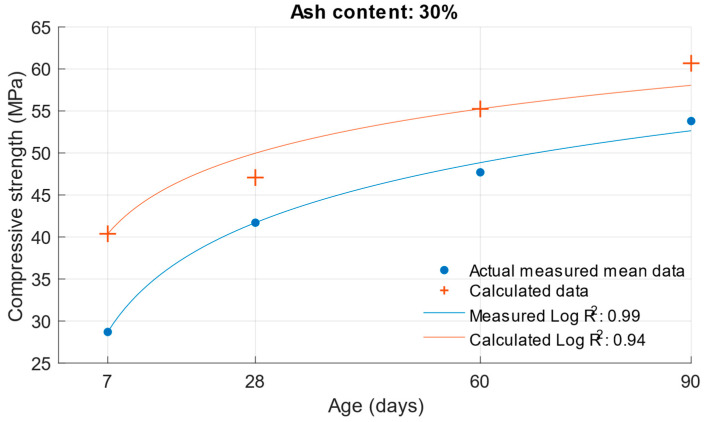
Difference between measured and calculated compressive strength with 30% fly ash.

The values in Figure 8, Figure 9, Figure 10 and Figure 11 demonstrate that the previous higher scattering of values for an age of 7 days could be confirmed. There was also a high variance for the calculated strengths of cement mortars with 30% cement replacement by fly ash. Table 5 shows the difference in the calculated k-values according to Equation (1). Based on these values, the previous assumption can be confirmed: that the largest deviations of the determined k-values would be for the age of 7 days and the 10% cement replacement with fly ash. For these k-values, there was a difference of up to 0.57. In Figure 12, Figure 13 and Figure 14 are shown differences between measured and calculated compressive strength with different amounts of fly ash. And in Table 6 are shown k-values for compressive strength calculated according to formula (1) and according to formula (6).

### 3.4. Determination of K-Value by the Depth of Penetration of Water Under Pressure

**Figure 15:**
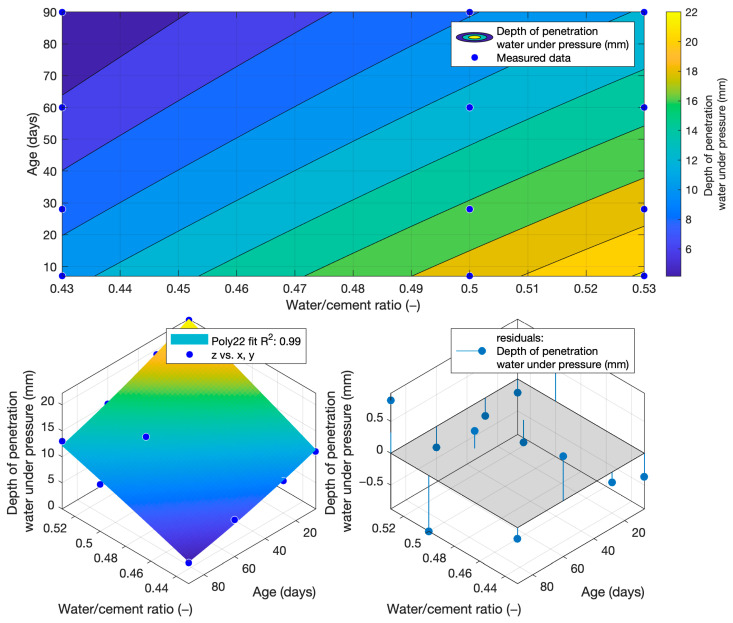
Function of the depth of penetration of water under pressure.

To determine the k-value using the depth of penetration of water under pressure, the dependence of this property on the water/cement ratio and the age of the cement mortars was constructed. This function is shown in Figure 15. Its coefficient of determination was equal to 0.99.

(7) $$f_{dw}\left(w,t\right)=-46.3633+156.5805\cdot w+0.032\cdot t-47.619\cdot w^{2}-0.3431\cdot w\cdot t+0.0003\cdot t^{2}$$

**Figure 16:**
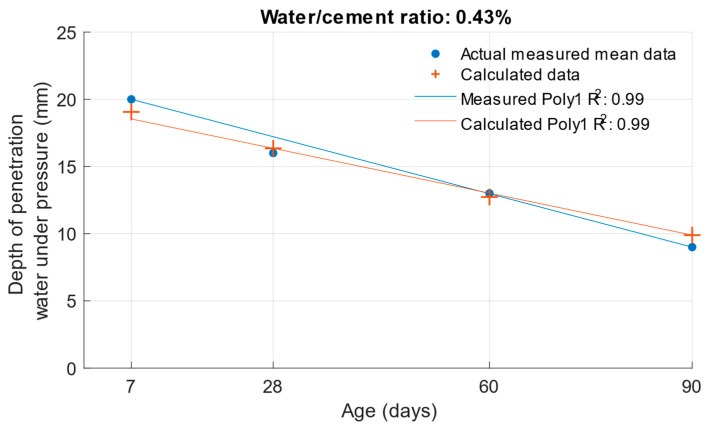
Difference between measured and calculated depths of penetration of water under pressure with water/cement ratio of 0.43.

**Figure 17:**
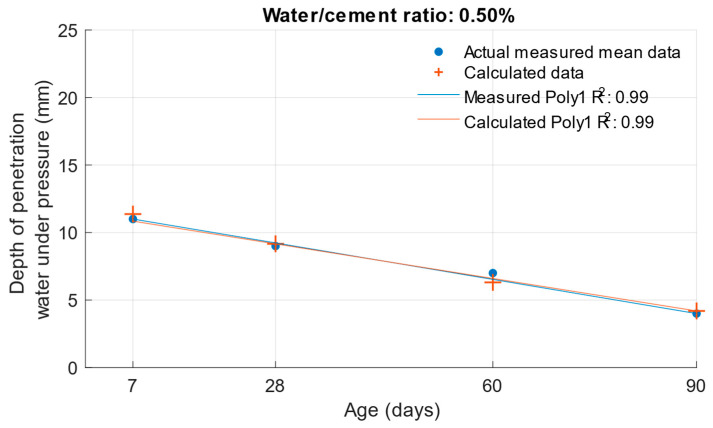
Difference between measured and calculated depths of penetration of water under pressure with water/cement ratio of 0.5.

**Figure 18:**
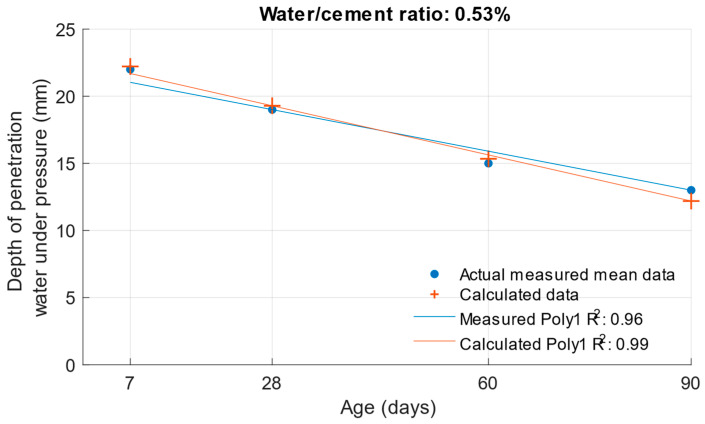
Difference between measured and calculated depths of penetration of water under pressure with water/cement ratio of 0.53.

The following graphs show the differences between the actual and calculated depth of penetration of water under pressure with different water/cement ratios. Figure 16 and Figure 17 show that for the water/cement ratios of 0.43 and 0.5, the largest difference was at the ages of 60 and 28 days. For a water/cement ratio of 0.53, shown in Figure 18, the largest difference between the calculated and measured values of depth of penetration of water under pressure was at an age of 90 days. The differences between the actual and calculated depth of penetration of water under pressure did not reach high values. The largest difference was a water/cement ratio of 0.5 at an age of 7 days, and this difference was only 0.94 mm. Numerical differences between measured and calculated depth of penetration of water under pressure with different water/cement ratios are shown in Table 7.

**Figure 19:**
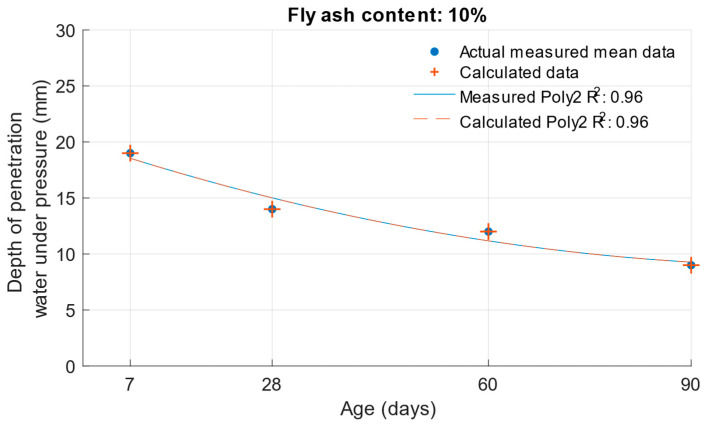
Difference between measured and calculated depths of penetration of water under pressure with 10% fly ash.

**Figure 20:**
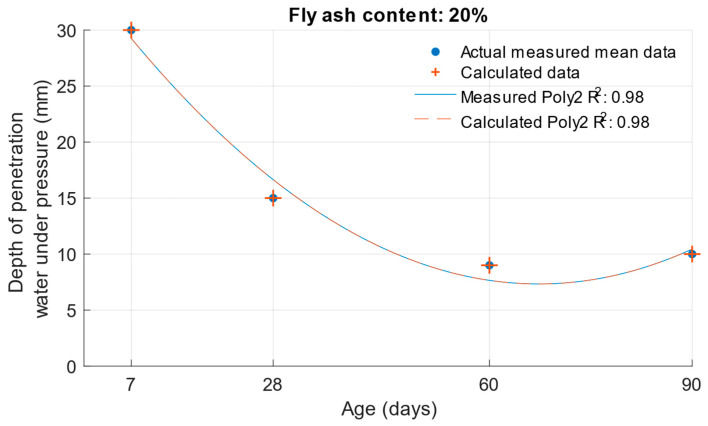
Difference between measured and calculated depths of penetration of water under pressure with 20% fly ash.

**Figure 21:**
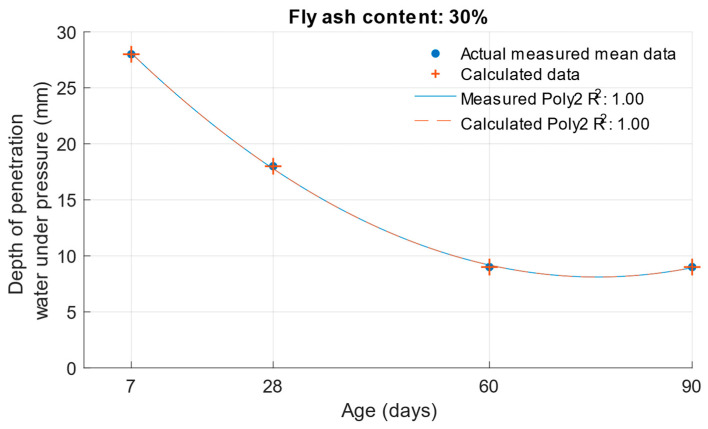
Difference between measured and calculated depths of penetration of water under pressure with 30% fly ash.

When calculating the depth of penetration of water under pressure for different cement substitutes with fly ash, the difference between the measured and calculated values was less than 0.01 mm. This corresponded to the high coefficient of determination of the depth of penetration of water under pressure function. Differences between measured and calculated depth of of pressurised water penetration with different fly ash content are shown in Figure 19, Figure 20 and Figure 21. Numerical differences between measured and calculated depth of penetration of water under pressure are shown in Table 8.

The k-values determined by the depth of penetration of water under pressure, shown in Table 9, reached a value of 1 or higher in the majority of cases. The water penetration depths given in Figure 6 for cement mortars with 10% fly ash were lower than the values of the reference cement mortar with the same water/cement ratio. This was why the k-values for this fly ash replacement were so high. For 20% and 30% cement replacements with fly ash, the k-values were equal to 1 and higher only at ages of 60 and 90 days.

### 3.5. Determination of K-Value by Depth of Carbonation

For carbonation depth, the determined function had the lowest coefficient of determination at only 0.8373. For this reason, the carbonation depth values used to determine the carbonation depth function were converted to the natural logarithm according to Equation (8). Since the carbonation depth results had a similar waveform to that of the exponential function, a high coefficient of determination was not achieved when using polynomial regression analysis. For this reason, a logarithmic transformation was used.

(8) $${dc}_{f}=\ln dc$$

Here, ${dc}_{f}$ is the carbonation depth used to determine the carbonation depth function in mm, and dc is the measured carbonation depth in mm.

**Figure 22:**
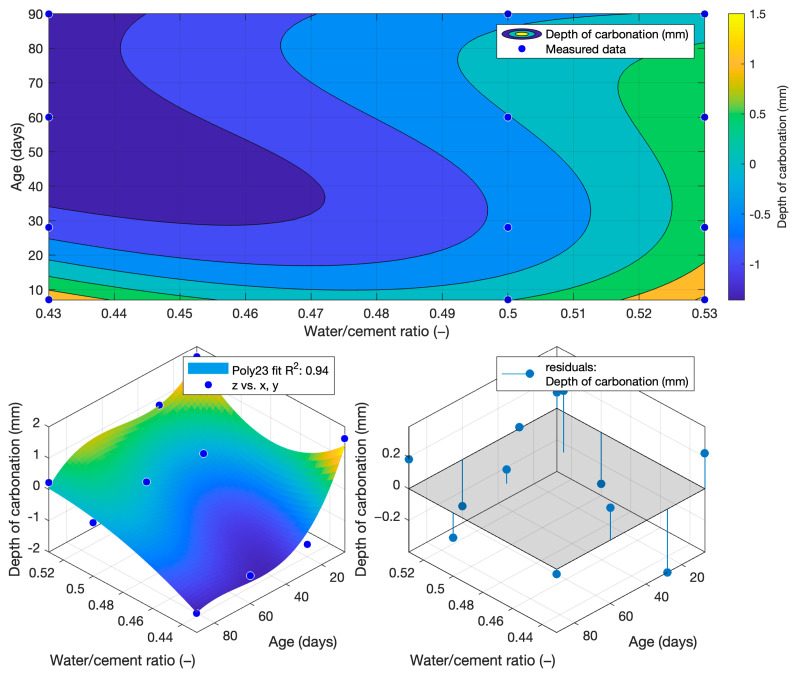
Function of depth of carbonation.

After converting the carbonation depths to a natural logarithm and then creating a carbonation depth function, the coefficient of determination increased to 0.94. The carbonation depth function is given in Equation (9) and Figure 22

(9) $$f_{dc}\left(w,t\right)=138.72-176.4\cdot w-0.15\cdot t+200.7\cdot w^{2}+0.43\cdot w\cdot t+0.001\cdot t^{2}-0.4\cdot w^{2}\cdot t-0.001\cdot w\cdot t^{2}$$

**Figure 23:**
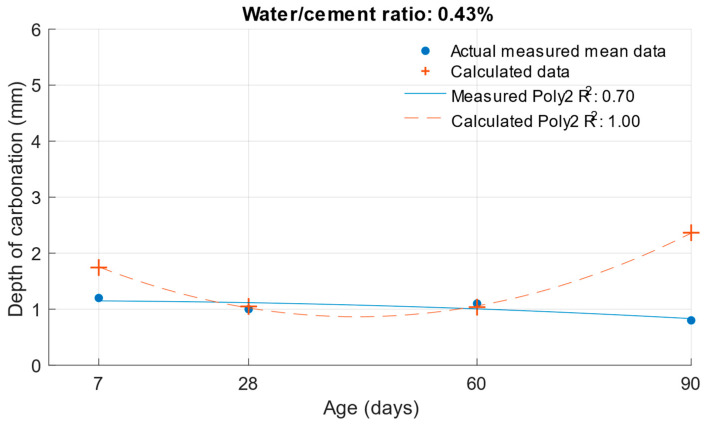
Difference between measured and calculated depths of carbonation with water/cement ratio of 0.43.

**Figure 24:**
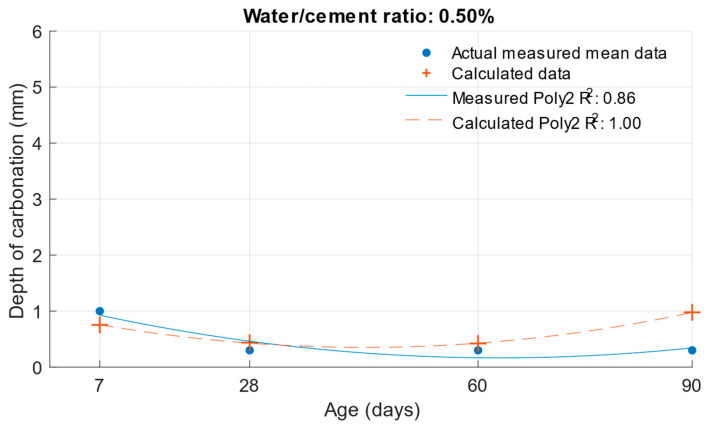
Difference between measured and calculated depths of carbonation with water/cement ratio of 0.50.

**Figure 25:**
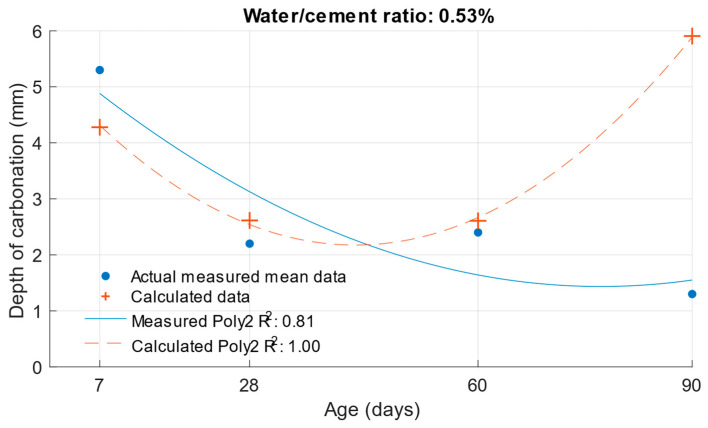
Difference between measured and calculated depths of carbonation with water/cement ratio of 0.53.

Figure 23, Figure 24 and Figure 25 illustrate the differences between the actual measured values and those calculated from the carbonation depth function, which were back-transformed by the exponential function after logarithmic transformation.

The differences between the measured and calculated carbonation depth values shown in Table 10 demonstrate that the highest deviations from the measured values were high for cement mortars. In some cases, the deviation was even more than 1 mm (these were also differences of more than 100% from the original measured value). It can be concluded that for these values, the k-values determined would be highly inaccurate.

**Figure 26:**
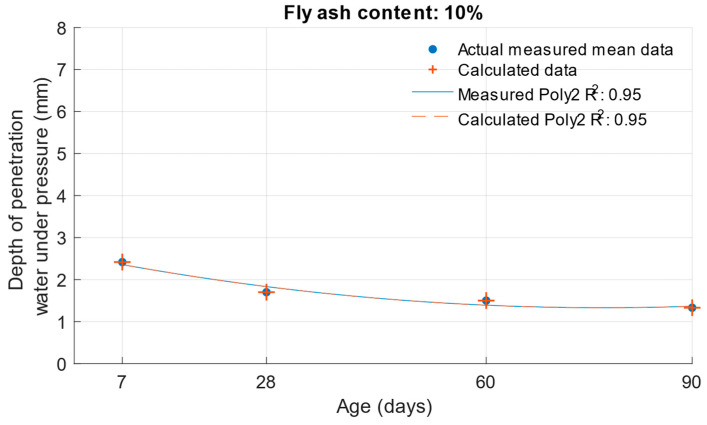
Difference between measured and calculated depths of carbonation with 10% fly ash.

**Figure 27:**
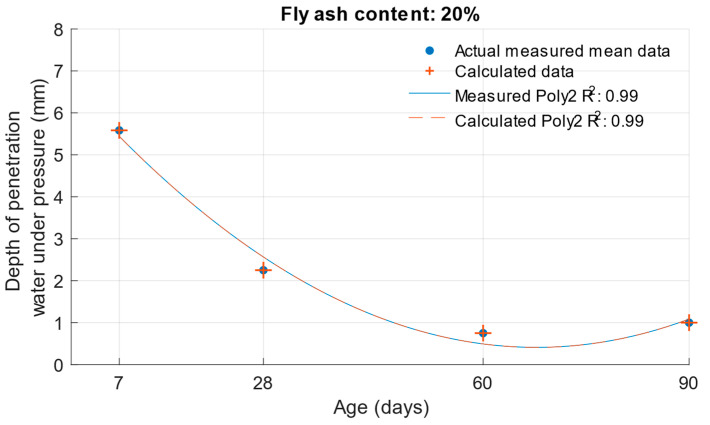
Difference between measured and calculated depths of carbonation with 20% fly ash.

**Figure 28:**
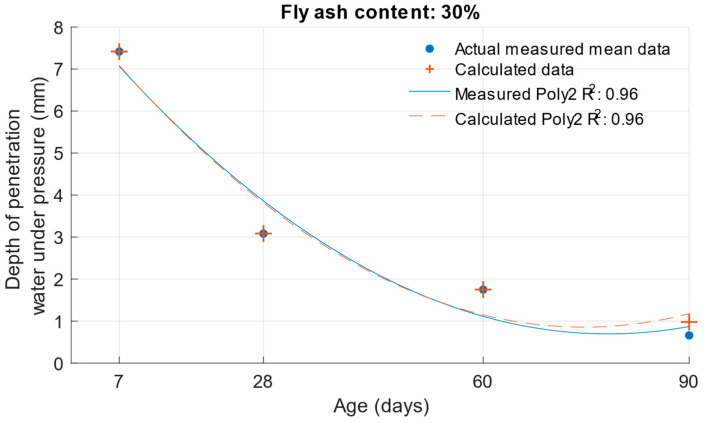
Difference between measured and calculated depths of carbonation with 30% fly ash.

The differences between the measured and calculated carbonation depths are shown in Figure 26, Figure 27 and Figure 28 and the specific numerical values in Table 11. In this case, the calculated values were also converted back to the original scale using an exponential function.

The differences in the carbonation depth values for the individual cement mortars with different cement replacements with fly ash were less than 0.01 mm in all cases except for the calculated carbonation depth at 90 days with 30% cement replacement with fly ash. In this case, the difference between the measured and calculated depth of carbonation was 0.24 mm.

Table 12 shows the k-values determined as a function of carbonation depth (9). From the differences obtained between the actual and calculated depth of carbonation shown in Table 10, it can be concluded that the determined k-values would not be as accurate as those for the depth of penetration of water under pressure. Although a high coefficient of determination was achieved, the differences in the carbonation depth values for the different water/cement ratios were quite high.

## 4. Discussion and Conclusions

The compressive strength results obtained with different water/cement ratios decreased with decreasing water/cement ratios. This was consistent with other results in [20,21]. Compressive strengths and activity indices were lower at early maturation ages and increased gradually as the age of cement mortars increased. This was consistent with the more gradual pozzolanic response of fly ash and the slower increase in the compressive strengths of these cement mortars [22,23].

The depth of penetration of water under pressure and the depth of carbonation of cement mortars increased as the water/cement ratio increased. This was primarily because higher water content in cement mortar causes higher porosity [24].

According to [25,26,27], fly ash negatively impacts the rate of carbonation, especially at early ages such as 7 and 28 days. At ages of 90 days and above, the depth of carbonation is reduced, but the same or lower carbonation depths are not achieved as they are for cement-only mixtures. This phenomenon could also be observed in this case, where the depths of carbonation at ages of 7 and 28 days with all fly ash replacements were greater than that of the reference cement mortar.

The k-values for power plant fly ash were determined according to the procedure described in Section 2. For the compressive strength, two procedures were used. The first was by using the compressive strength equation taken from the literature [11], and the second was by determining the dependence of the property on the water/cement ratio and the age of the cement mortar. Table 6 illustrates the disparity between the two procedures, highlighting the considerable differences in k-values in some cases.

One of the factors affecting the resulting k-value may have been the rounding of the results. However, in the case of both the compressive strength and depth of water penetration, the rounding was lower than required by the relevant standard for these tests. In the case of carbonation depth, the results were rounded according to the standard. Therefore, rounding had a low effect on the inferred k-values.

The k-values determined by compressive strength were lowest at 7 days of age for both methods used and increased with an increasing age up to 0.94 and 1.22 for 10 and 30% fly ash at 90 days of age. These results corresponded with [13] and as the amount of fly ash in the cement mortar increased, the k-values decreased. There was also a high variance for the calculated strengths of cement mortars with 30% cement replacement by fly ash. Table 5 shows the differences in the calculated k-values according to Equation (1).

The k-values determined by the depth of penetration of water under pressure reached a value of 1 or higher in the majority of cases. The water penetration depths given in Figure 6 for cement mortars with 10% fly ash were lower than the values of the reference cement mortar with the same water/cement ratio. This was why the k-values for this fly ash replacement were so high. For 20% and 30% cement replacement with fly ash, the k-values were equal to 1 and higher only at ages of 60 and 90 days.

From the high k-values for the depth of pressurized water penetration, it is possible to conclude that higher amounts of fly ash could be used as a cement replacement for exposure classes XC and XD. However, it is important to consider that for these exposure classes, EN 206+A2 specifies a requirement for compressive strength and the equivalent binder quantity [8]. Therefore, in order to determine the optimum k-value, it is necessary to take into account the k-values for the determination using other properties and from these, for example, use a weighted average to determine the optimum k-value for a given amount of admixture and age.

In the case of k-value determination using carbonation depth, the k-value again increased with increasing age. The highest k-values were obtained for 20% replacement of cement with fly ash at 60 and 90 days of age, when the k-values were higher than 1. These result corresponded to those in [13].

EN 206+ A2 does not take into account the effect of age on the k-value and only gives fixed values for fly ash [8]. From the results obtained, it can be said that it would be advisable to take this effect into account in the design of concrete. In some cases, the concrete performance requirements are given after 90 days, in which case the use of a k-value of 0.4 for a maximum of 30% fly ash as given in EN 206+ A2 does not allow the effective use of fly ash. In this case, the k-value is more than 0.2 higher than the normal value and almost 0.3 higher when using the 90-day k-value. After a simple calculation of the equivalent amount of binder with k-values of 0.4, 0.63, and 0.67 while maintaining 350 kg of binder, we should achieve the same properties with a k-value of 0.4 using 310 kg of cement, with a k-value of 0.63 using 290 kg of cement, and with a k-value of 0.67 using 280 kg of cement. From this it can be said that using the optimum k-value for a given age can save up to 70 kg of cement per 1 m^3^ of concrete.

If we consider that the k-value is directly based on the activity indices (higher activity index means a higher k-value), then there should be a strong positive relationship between the activity index and the k-value (high positive correlation coefficient). If we calculate the correlation coefficient between the activity index and the k-value determined by Equation (1), we obtain a value of 0.95. This result implies a strongly positive dependence between these two values. Calculating the correlation coefficient between the activity index and the k-value calculated using the optimisation problem, we obtain a value of 0.67. In this case, the dependence is not as ‘strong’ as for the k-value computed using Equation (1), and these k-values are not as accurate as the k-values constructed using relation (1).

If we proceed in the same way for the depth of penetration of water under pressure and the depth of carbonation, it is necessary to use an inverse proportionality between the ‘efficiency’ of the fly ash and the given property to calculate the ‘efficiency index’ (e.g., the lower depth of penetration of water under pressure is, the higher the ‘efficiency’ of the fly ash will be). Calculating the correlation coefficient for the depth of seepage through pressurised water and the k-value for this property gives a value of 0.84. Thus, the correlation between these values is high, and the k-values can be considered as corresponding to reality. For the depth of carbonation, the correlation coefficient was determined to be only 0.53 in this study. In this case, the k-values could be considered less accurate.

When determining the k-value using an optimisation problem based on the functions of individual properties dependent on the water/cement ratio and the age of the cement mortars, the first inaccuracies in the k-value occur when determining the functions. Of course, the coefficient of determination and the difference between the measured values and the values calculated using these functions have the greatest influence. When determining the k-value, a value equal to one is taken as the initial k-value. Therefore, the calculated values of the individual features are the same for each age since the only difference is in the age, and the water/cement ratio is 0.50 for all cases. The k-value is then determined so that the difference between the calculated and measured properties is as small as possible. Thus, the k-value itself is directly affected by the difference between the measured and calculated k-value of the property. Therefore, the calculation of the k-value theoretically ‘makes up’ the difference between the measured and calculated value. It follows that if the function is accurate and the differences between the measured and calculated values of the property for different water/cement ratios are low, the k-value is unaffected by the error that arises from the difference between the measured and calculated k-values, but it only takes into account the effect of the fly ash on the property.

Increasing the coefficients of determination and reducing the differences between measured and calculated property values is possible in two ways: by increasing the number of measurements, thus increasing the statistical reliability, or by finding the most appropriate mathematical function that would most accurately describe the evolution of the property as a function of the water/cement ratio and age. In our case, for the compressive strength and the depth of penetration of water under pressure, it would be appropriate to increase the number of measurements and thus increase the statistical reliability of the function. Regarding compressive strength, there were higher deviations between measured and calculated compressive strengths for different water/cement ratios and different cement substitutes for fly ash. Therefore, some of the calculated k-values were entirely irrelevant in this way. Concerning pressurised water seepage depth, both a high coefficient of determination and a low difference between the calculated and measured depths of penetration of water under pressure were achieved. Therefore, the k-values could be considered relevant for the depth of penetration of water under pressure. For the depth of carbonation, although a high coefficient of determination was achieved, significant differences between the measured and calculated values were obtained. In this case, it would be best to change the carbonation depth testing procedure. Using a different method of determination or potentially increasing the carbon dioxide concentration during accelerated carbonation testing would result in much higher carbonation depths. This would prevent low carbonation values that have a high percentage deviation (even with a small deviation in millimetres (e.g., 0.5 mm) from the measured values (for 0.5 mm, even 50% difference from the measured values) and thus achieve lower percentage deviations of values for different carbonation depths with different water/cement ratios.

From a practical point of view, it is, therefore, possible to use this method to consider the influence of active admixtures on the durability of cement mortars and concretes and, thus, make their use in construction practice more efficient. However, since it is currently more common in practice to use blended cements of the CEM II type, it would be advisable to extend the methodology to include the possibility of using a combination of these cements and active admixtures, particularly concerning the optimum amount of active admixture, so that the mechanical and durability properties of these concretes are not significantly impaired.

## Figures and Tables

**Table 1:** Physical properties of fly ash and cements.

Property	Fly Ash Opatovice	CEM I 42.5 R Mokrá
Specific surface area [cm^2^/g]	4705	3885
Specific gravity [g/cm^3^]	2.75	3.15

**Table 2:** Chemical compositions of cements and fly ash.

	LOI [%]	Chlorides [%]	SO_3_ [%]	SiO_2_ [%]	Al_2_O_3_ [%]	CaO [%]	Fe_2_O_3_ [%]	Na_2_O [%]	K_2_O [%]	MgO [%]
Fly Ash Opatovice	1.24	<0.0100	0.44	52.7	30.6	1.99	10.6	-	-	-
CEM I 42.5 R	4.07	0.050	3.2	19.5	4.8	62.5	3.3	0.11	0.74	1.41

**Table 3:** Cement mortars: mix proportions.

Recipe [kg/m^3^]	CEM I 42.5 R	Water	Fly Ash	Sand 0.1–0.6 mm	Sand 0.6–1.2 mm	Sand 1–4 mm	Water/Cement Ratio ^1^
R-I-1	511	254	-	443	517	572	0.5
R-I-2	511	220	-	443	517	572	0.43
R-I-3	511	271	-	443	517	572	0.53
A-I-10	460	254	51	443	517	572	0.5
A-I-20	409	254	102	443	517	572	0.5
A-I-30	358	254	153	443	517	572	0.5

^1^ For cement mortars with fly ash, the water/cement ratio was calculated with a k-value equal to one.

**Table 4:** Differences between calculated and measured compressive strength with different water/cement ratios.

Water/Cement Ratio [-]	Age [Days]	Measured [MPa]	Calculated [MPa]	Difference [MPa]
0.43	7	52.70	56.05	−3.35
	28	64.60	62.34	2.26
	60	73.10	69.88	3.22
	90	73.90	74.72	−0.82
0.5	7	37.30	40.38	−3.08
	28	48.90	47.08	1.82
	60	53.30	55.25	−1.95
	90	60.30	60.68	−0.38
0.53	7	38.60	37.38	1.22
	28	49.40	44.26	5.14
	60	51.70	52.70	−1.00
	90	57.70	58.38	−0.68

**Table 5:** Differences between calculated and measured compressive strength with different percentages of fly ash.

Percentage of Fly Ash	Age [Days]	Measured [MPa]	Calculated [MPa]	Difference [MPa]
10	7	30.30	36.59	−6.29
	28	50.00	50.00	0.00
	60	52.80	52.80	0.00
	90	59.90	59.90	0.00
20	7	31.30	36.59	−5.29
	28	45.20	45.20	0.00
	60	53.30	53.30	0.00
	90	63.10	63.10	0.00
30	7	28.70	36.59	−7.89
	28	41.70	43.61	−1.91
	60	47.70	52.24	−4.54
	90	53.80	58.07	−4.27

**Table 6:** K-values according to compressive strength.

k-Value [-]				
Mixture	Age [Days]	From the Formula (1)	From Regression (6)	Difference
A-I-10	7	0.57	0.00	0.57
A-I-20		0.54	0.50	0.04
A-I-30		0.43	0.67	−0.24
A-I-10	28	0.87	1.39	−0.52
A-I-20		0.72	0.84	−0.12
A-I-30		0.63	0.68	−0.05
A-I-10	60	0.92	0.47	0.45
A-I-20		0.98	0.81	0.17
A-I-30		0.71	0.70	0.01
A-I-10	90	0.94	0.85	0.09
A-I-20		1.22	1.19	0.03
A-I-30		0.67	0.72	−0.05

**Table 7:** Difference between calculated and measured depths of pressurised water penetration with different water/cement ratios.

Water/Cement Ratio [-]	Age [Days]	Measured [mm]	Calculated [mm]	Difference [mm]
0.43	7	11.00	11.37	−0.37
	28	9.00	9.16	−0.16
	60	7.00	6.31	0.69
	90	4.00	4.19	−0.19
0.50	7	20.00	19.06	0.94
	28	16.00	16.35	−0.35
	60	13.00	12.73	0.27
	90	9.00	9.89	−0.89
0.53	7	22.00	22.21	−0.21
	28	19.00	19.29	−0.29
	60	15.00	15.34	−0.34
	90	13.00	12.19	0.81

**Table 8:** Differences between calculated and measured depths of penetration of water under pressure with different percentages of fly ash.

Percentage Of Fly Ash	Age [Days]	Measured [mm]	Calculated [mm]	Difference [mm]
10	7	19.00	19.00	0.00
	28	14.00	13.99	0.01
	60	12.00	12.00	0.00
	90	9.00	8.99	0.01
20	7	30.00	29.99	0.01
	28	15.00	15.00	0.00
	60	9.00	9.00	0.00
	90	10.00	10.00	0.00
30	7	28.00	27.99	0.01
	28	18.00	18.00	0.00
	60	9.00	9.00	0.00
	90	9.00	9.00	0.00

**Table 9:** K-values according to depths of pressurised water penetration.

	K-Value According to Depth of Pressurised Water Penetration [-]			
Age [Days]	7	28	60	90
A-I-10	1.01	1.49	1.17	1.23
A-I-20	0.11	1.14	1.45	0.99
A-I-30	0.50	0.89	1.30	1.08

**Table 10:** Differences between calculated and measured depths of carbonation with different water/cement ratios.

Water/Cement Ratio [-]	Age [Days]	Measured [mm]	Calculated [mm]	Difference [mm]
0.43	7	1.17	1.64	−0.47
	28	1.00	1.07	−0.07
	60	1.08	1.01	0.07
	90	0.75	1.84	−1.09
0.50	7	1.00	0.78	0.22
	28	0.33	0.48	−0.14
	60	0.33	0.42	−0.09
	90	0.25	0.76	−0.51
0.53	7	5.33	4.07	1.26
	28	2.17	2.66	−0.50
	60	2.42	2.49	−0.07
	90	1.25	4.40	−3.15

**Table 11:** Differences between calculated and measured depths of carbonation with different percentages of fly ash.

Percentage of Fly Ash	Age [Days]	Measured [mm]	Calculated [mm]	Difference [mm]
10	7	2.42	2.42	0.00
	28	1.70	1.70	0.00
	60	1.50	1.50	0.00
	90	1.33	1.33	0.00
20	7	5.58	5.58	0.00
	28	2.25	2.25	0.00
	60	0.75	0.75	0.00
	90	1.00	1.00	0.00
30	7	7.42	7.42	0.00
	28	3.08	3.08	0.00
	60	1.75	1.75	0.00
	90	0.66	1.00	−0.24

**Table 12:** K-values according to depths of carbonation.

	K-Value According to Depth of Carbonation			
Age [Days]	7	28	60	90
A-I-10	0.76	0.67	0.74	1.61
A-I-20	0.65	0.76	1.15	1.67
A-I-30	0.73	0.78	0.88	1.54

## Data Availability

The original contributions presented in this study are included in the article. Further inquiries can be directed to the corresponding author.
